# Case Report: An Atypical Angelman Syndrome Case With Obesity and Fulfilled Autism Spectrum Disorder Identified by Microarray

**DOI:** 10.3389/fgene.2021.755605

**Published:** 2021-09-22

**Authors:** Areerat Hnoonual, Phawin Kor-anantakul, Chariyawan Charalsawadi, Juthamas Worachotekamjorn, Pornprot Limprasert

**Affiliations:** ^1^ Department of Pathology, Faculty of Medicine, Prince of Songkla University, Songkhla, Thailand; ^2^ Center of Excellence for Medical Genomics, Medical Genomics Cluster, Department of Pediatrics, Faculty of Medicine, Chulalongkorn University, Bangkok, Thailand; ^3^ Excellence Center for Genomics and Precision Medicine, King Chulalongkorn Memorial Hospital, Thai Red Cross Society, Bangkok, Thailand; ^4^ Department of Pediatrics, Faculty of Medicine, Prince of Songkla University, Songkhla, Thailand; ^5^ Faculty of Medicine, Siam University, Bangkok, Thailand

**Keywords:** angelman syndrome, autism, methylation-specific PCR, microarray, uniparental disomy

## Abstract

Autism spectrum disorder (ASD) is a group of neurodevelopmental disorders which are etiologically heterogeneous. Chromosomal microarray is now recommended as the first-tier clinical diagnostic test for ASD. We performed chromosomal microarray in 16 Thai patients with ASD using an Illumina HumanCytoSNP-12 v2.1 array and found one case with uniparental disomy (UPD) of chromosome 15. Methylation-specific PCR showed abnormal methylation of the maternal *SNRPN* allele. Haplotype analysis revealed that the patient had received both chromosomes 15 from his father. These results were consistent with Angelman syndrome. However, his clinical features had no clinical significance for classic Angelman syndrome. He had first presented at the pediatric clinic with no speech, poor social interaction skills and repetitive behaviors consistent with ASD based on the DSM-IV criteria at 2 years of age and later confirmed by ADOS at 5 years of age. He was strikingly overweight but had no dysmorphic facies, seizures nor ataxia and was diagnosed as non-syndromic ASD, a diagnosis which was believed until at 10 years of age, his DNA was included for analysis in this current cohort study. Our findings suggest that ASD patients with unknown etiology should be considered for methylation-specific PCR testing for Angelman syndrome where chromosomal microarray is not available. In the study, we also review the clinical features of Angelman syndrome caused by UPD and the frequency of ASD in individuals with Angelman syndrome.

## Introduction

Autism spectrum disorder (ASD) is a heterogeneous group of neurodevelopmental disorders characterized by impaired social interaction and communication, and stereotyped behaviors and interests. Currently, a number of genetic syndromes are known to be related to ASD. Abnormalities of chromosome 15 are the most frequently reported in ASD, especially 15q11-q13 duplications which have been reported to occur in 0.25–3% of ASD cases depending on sample ascertainment (Cook et al., 1997; Schroer et al., 1998; Depienne et al., 2009; Sanders et al., 2015). Chromosome 15q11-q13, the Prader-Willi/Angelman syndrome (PWS/AS) critical region, contains a number of imprinted and non-imprinted genes including the *UBE3A* and GABA_A_ receptor subunit genes that may be associated with the presence of ASD. Several association studies of these genes have found significant associations with autism (Cook et al., 1997; Cook et al., 1998; Buiting et al., 2016; Khatri and Man, 2019).

Angelman syndrome (AS) is a neurogenetic disorder characterized by severe developmental delay usually noticeable at 6–12 months of age. The notable clinical features include developmental delay, severe speech delay with minimal to no use of word, ataxia, hand flapping, happy demeanor and inappropriate laughter or smiling. Other clinical features associated with AS include microcephaly and seizure with a characteristic abnormal electroencephalogram (EEG) pattern with large amplitude slow-spike waves. Irregular sleep-wake cycles, wide-spaced teeth, frequent drooling and hypopigmentation are also frequently observed (Williams et al., 2006). AS is caused by four known genetic mechanisms including deletion of chromosome 15q11-q13, mutations of *UBE3A*, uniparental disomy (UPD) of chromosome 15 and imprinting center defect (Buiting et al., 2016; Beygo et al., 2019). While the loss of function of the maternally inherited *UBE3A* gene causes AS, abnormalities of chromosome 15q (deletions/duplications) that contain the *UBE3A* gene have been reported in association with ASD (Cook et al., 1997; Depienne et al., 2009; Vatsa and Jana, 2018; Khatri and Man, 2019), suggesting that the *UBE3A* gene may also be implicated in autism. AS has a high comorbidity with autism and shows many overlapping clinical features of autism (Steffenburg et al., 1996; Peters et al., 2004; Trillingsgaard and ØStergaard, 2004; Bonati et al., 2007). AS is also considered as a syndromic form of ASD. However, previous studies have shown a wide range of ASD prevalence in patients with AS (Chan et al., 1993; Steffenburg et al., 1996; Peters et al., 2004; Trillingsgaard and ØStergaard, 2004; Sahoo et al., 2006; Bonati et al., 2007; Peters et al., 2012; Moss et al., 2013; Mertz et al., 2014; Wink et al., 2015) but there are very few studies reporting on the prevalence of AS in patients with ASD (Schroer et al., 1998; Depienne et al., 2009). Several recent studies have suggested that ASD is strongly associated with genetic variations, including copy number variations (CNVs). Chromosomal microarray (CMA) has enabled identification of CNVs with a much higher resolution than standard chromosome analysis. In addition, single nucleotide polymorphism (SNP) arrays based on CMA can also detect copy number neutral abnormalities such as regions of long contiguous stretches of homozygosity (LCSH) and UPD which are associated with an increased risk of genetic diseases including PWS and AS. CMA is now recommended as the first-tier clinical diagnostic test for individuals with ASD, developmental delay/intellectual disability, and multiple congenital anomalies of unknown causes (Miller et al., 2010; Battaglia et al., 2013; Schaefer et al., 2013).

In this study, we aimed to identify novel or pathogenic CNVs in selected Thai children with ASD, and we found a patient with atypical AS who had originally been diagnosed as ASD. SNP arrays identified the absence of heterozygosity (AOH) along the long (q) arm of chromosome 15. Subsequently, methylation-specific PCR (MS-PCR) analysis confirmed the AS diagnosis in this patient. A haplotype analysis of chromosome 15 showed that the patient had inherited both chromosomes 15 from his father, indicating paternal UPD consistent with a diagnosis of AS. In addition, we studied 100 patients with ASD for PWS/AS methylation-specific PCR screening, and no other patients with AS or PWS were detected in our ASD cohort.

## Case Presentation

A 2-year-old boy initially presented with no speech, poor social interaction skills and repetitive behaviors consistent with ASD based on the DSM-IV criteria, which also fulfilled the DSM-V criteria. The patient was the second child born to healthy and unrelated Thai parents. He had one older sister with normal development. His birth weight was 3,600 g with no perinatal complications. He first stood with support at 10 months, but did not walk without support until the age of 27 months. He was unable to hold a spoon and feed himself until 45 months of age. He never developed verbal language skills. His growth curves were within normal ranges, including head circumference, except he had been overweight since 2 years of age (Supplementary Figure S1). His BMI at 10 years was 29.7 kg/m^2^ (>97th centile, Z score = 3.79). He had no hypopigmentation, and had never shown signs of seizure, ataxia or hypotonia. He usually walked on tip toes. He had poor eye contact and had a repetitive behavior in constantly spinning the wheels of a toy car. He engaged in hand-flapping whenever he was happy. He had no self-injury nor aggressive behaviors. He liked to bite things (i.e., clothes, drinking straws). At the age of 5 years, he was assessed using the Autism Diagnosis Observation Schedule (ADOS)-Module 1 by a developmental pediatrician. His scores for communication were 4 (cutoff = 4), 8 for reciprocal social interaction (cutoff = 7), and 1 for repetitive behaviors (Supplementary Table S1). The scores were consistent with a diagnosis of autistic disorder. At 10 years of age, he had a non-verbal IQ rating of 42 (Stanford Binet Intelligence Scales-Fifth edition) and the Vineland Adaptive Behavior Scales-II showed moderate deficits in all three domains, communications, daily living skills and socialization. We also observed that his autistic features evolved as his social interaction skills improved over time. For example, at 11 years of age, he liked to play with his peers and parents, although he still could not speak understandable words.

Initially, he was tested for common genetic causes of ASD, which revealed normal karyotyping and normal CGG repeats on the *FMR1* gene. Electroencephalogram (EEG) was also normal. Following the recommendations from the International Standard Cytogenomic Array (ISCA) and the American College of Medical Genetics (ACMG) for the use of CMA, he was screened with the CMA at 10 years of age to look for pathogenic CNVs. Written informed consent was obtained from the patient’s parents for publication of this case report.

## Materials and Methods

### Patients

A total of 16 patients with ASD of unknown cause were recruited from a large cohort of Thai children with non-syndromic ASD reported in a previous study (Charalsawadi et al., 2014). The patients fulfilled the criteria for ASD diagnosis according to the Diagnostic and Statistical Manual of Mental Disorders, Fourth Edition (DSM-IV) (Hansakunachai et al., 2014). Karyotyping and Fragile X DNA testing had been done in all patients and also *MECP2* sequencing testing in females as initial tests and all tests had shown normal results. In addition, we performed PWS/AS methylation specific PCR on 100 patients with ASD to screen for AS and PWS to find the frequency of PWS/AS in ASD patients.

### Chromosomal Microarray Analysis

SNP arrays were performed for the 16 patients with unexplained ASD using a HumanCytoSNP-12 DNA Analysis BeadChip v2.1 kit (Illumina, San Diego, California, United States), which contains approximately 3,00,000 SNP markers. The results were analyzed by BlueFuse Multi software and GenomeStudio Data Analysis Software v. 2011.1 based on the reference human genome (hg19/GRCh37). The data were then interpreted based on guidelines from the ISCA and ACMG (Miller et al., 2010; Kearney et al., 2011; Riggs et al., 2020).

### Methylation-specific Polymerase Chain Reaction (MS-PCR) Analysis

The DNA samples were treated with bisulfate using a process described previously (Kubota et al., 1997) with modifications (Supplementary Material). The modified DNA sample was then used as a template in the following MS-PCR. MS-PCR analysis of the *SNRPN* gene was performed using original and alternative primers (Kubota et al., 1997; Hussain Askree et al., 2011) (Supplementary Table S2). Multiplex PCR was performed in a total volume of 10 µL containing 50 ng modified DNA template, 1X PCR buffer, 1.5 mM MgCl_2_, 0.2 mM dNTPs and 0.6 units of FastStart Taq DNA polymerase (Roche Applied Science). PCRs were carried out by denaturation at 96°C for 10 min, followed by 35 cycles at 94°C for 30 s, 64°C for 30 s, 72°C for 1 min, and final extension at 72°C for 10 min. The PCR products were run on 2.5% agarose gel electrophoresis and visualized by staining with ethidium bromide.

### Fluorescence *in situ* Hybridization (FISH)

FISH was performed to check specifically for chromosome 15 deletions, according to the manufacturer’s instructions with minor modifications. The DNA probes used in the FISH analysis included a triple probe mix of Vysis Prader-Willi/Angelman Region Probe - LSI D15S10 SpectrumOrange at 15q11.2, CEP 15 (D15Z1) SpectrumAqua at 15p11.2, and PML SpectrumGreen Probe at 15q22 (Abbott Molecular Inc.). The FISH images were captured and analyzed using the Isis program (MetaSystems).

### Haplotype Analysis of UPD

To investigate either paternal or maternal UPD in the proband with autism, six microsatellite markers (D15S1012, D15S643, D15S983, D15S979, D15S657, and D15S966) located along the q arm of chromosome 15 were selected from the GenBank database (Supplementary Table S3). The details of haplotype analysis using capillary electrophoresis are shown in Supplementary Material.

## Results

### CMA, MS-PCR Analysis and FISH

**FIGURE 1:**
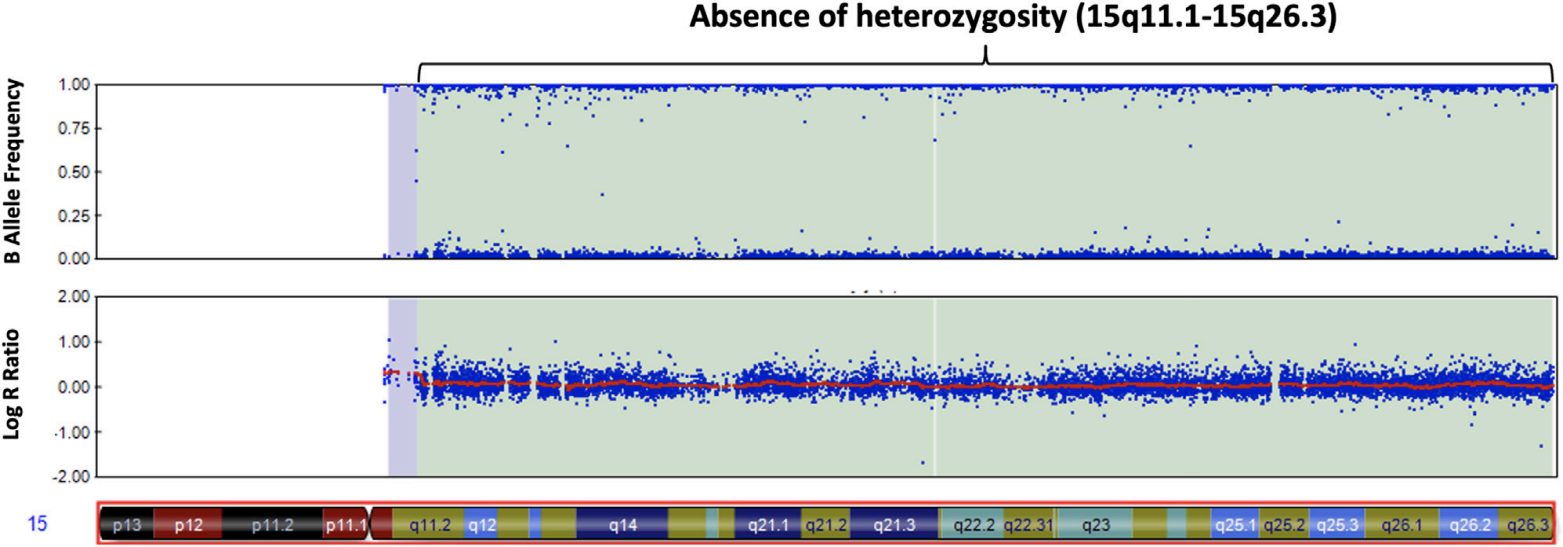
Chromosomal microarray analysis of our patient with autism. The SNP microarray shows an absence of heterozygosity along the q arm of chromosome 15 in the B allele frequency plot **(upper panel)**. The Log R ratio plot **(lower panel)** shows no change in copy number in the q arm of chromosome 15.

**FIGURE 2:**
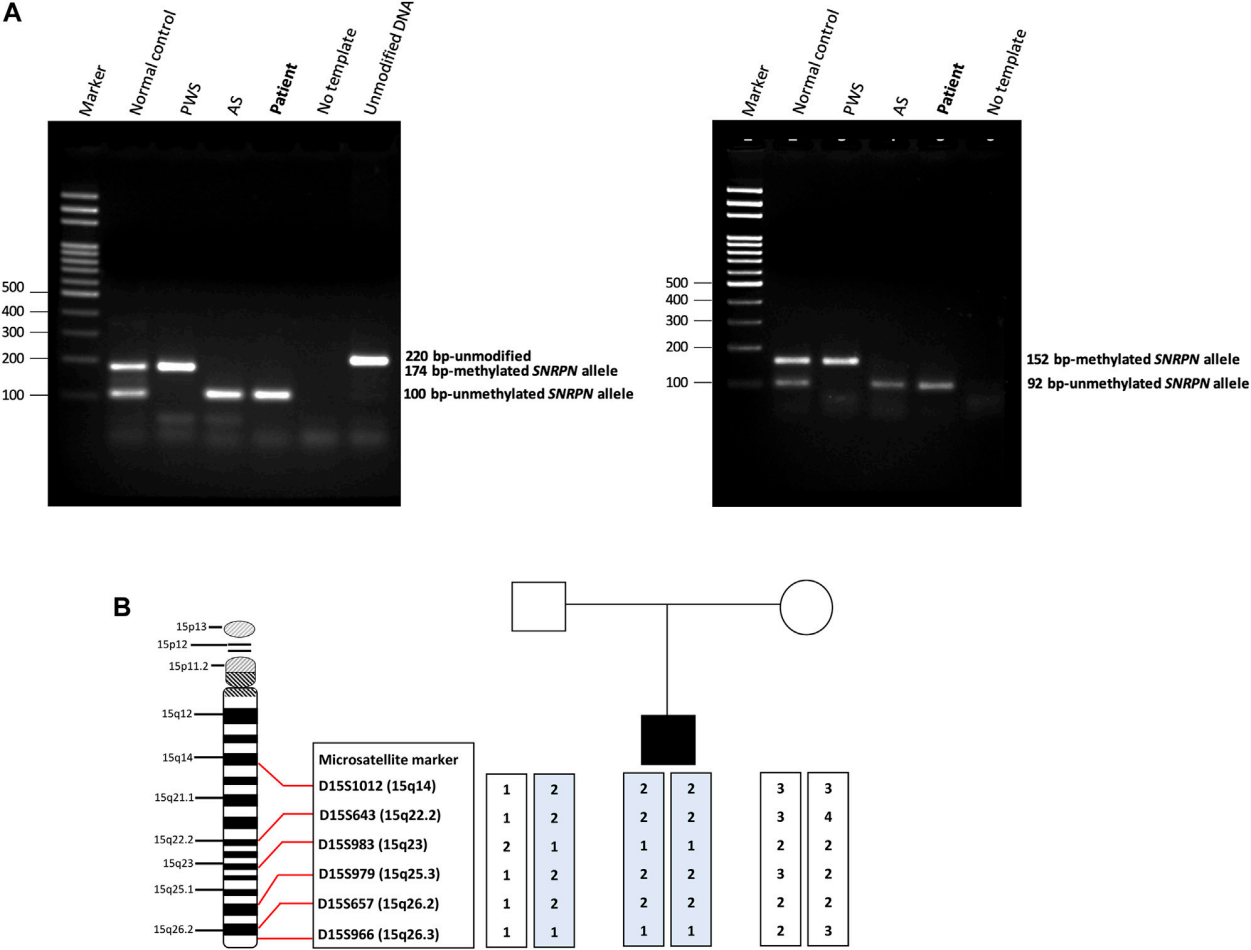
Methylation-specific PCR (MS-PCR) results and haplotype analysis. **(A)** The **(left panel)** shows the results of MS-PCR analysis of a patient using an original primer set designed by Kubota et al. The MS-PCR results from a normal control show a 100-bp PCR product representing the unmethylated paternal allele and a 174-bp PCR product representing the methylated maternal allele. Consistent with the clinical diagnosis of AS, our patient showed only a 100-bp PCR product derived from the unmethylated paternal allele. The **(right panel)** shows the results of MS-PCR analysis of our patient using an alternative primer set designed by Hussain Askree et al. A normal control shows a 92-bp PCR product of the unmethylated paternal allele and a 152-bp PCR product of the methylated maternal allele. Our patient showed only a 92-bp PCR product derived from the unmethylated paternal allele, consistent with a diagnosis of AS. AS, Angelman syndrome; PWS, Prader-Willi syndrome. **(B)** Haplotype analysis of the patient and his parents. The results of a haplotype analysis using six microsatellite markers across the q arm of chromosome 15 revealed that the patient had inherited both chromosomes 15 from his father. It also showed isodisomy which indicated the most likely cause to be a meiosis II nondisjunction event.

We screened 16 patients with unexplained ASD for genomic imbalances and SNP array analysis in a 10-year-old boy with ASD detected an absence of heterozygosity (AOH) region of 82.2 Mb (15q11.1–15q26.3), possibly indicative of UPD (Figure 1). No pathogenic CNVs were detected in the other 15 patients. To validate the results of the microarray and to investigate the possible alterations of the *SNRPN* methylation status, MS-PCR analyses with both original and alternative primer sets were further performed, which found the absence of a maternal methylation of the *SNRPN* gene in the proband, which was consistent with a diagnosis of AS (Figure 2A). FISH testing confirmed that the patient did not carry deletions on chromosome 15 (Supplementary Figure S2) and chromosome analysis using G-banding in this patient showed a normal karyotype, and thus he was suspected of having UPD.

### Haplotype Analysis of UPD

Haplotype analysis with six microsatellite markers of the q arm of chromosome 15 was then used to investigate the genetic mechanisms of AS in the patient. The results confirmed that the patient had received both chromosomes 15 from his father, indicating that paternal UPD was the likely cause of AS in this patient. In addition, both paternally-derived alleles showed the same haplotypes, indicating paternal isodisomy of chromosome 15. The results of the haplotype analysis are shown in Figure 2B.

### Screening of PWS and AS in Patients With ASD

Because of the unexpected result of detecting AS in the patient with ASD, we further screened a total of 100 ASD patients to look for PWS/AS in our ASD patient’s cohort using MS-PCR analysis. In this group, all of the patients had normal paternal and maternal methylation of the *SNRPN* gene, indicating that none of them had PWS or AS, suggesting that PWS/AS is not a common genetic cause of patients with ASD. The workflow and summary of the results of this study are shown in Supplementary Figure S3.

## Discussion

We identified an AS patient with ASD caused by paternal UPD of chromosome 15. The patient was first diagnosed with ASD of unknown cause and a chromosomal microarray was performed following the ACMG and ISCA recommendations. The microarray results revealed UPD of chromosome 15. The MS-PCR analysis of this patient showed abnormal methylation of the maternal *SNRPN* allele and haplotype analysis showed that the patient had paternal uniparental isodisomy, resulting from meiosis II nondisjunction. Therefore, the results of all molecular genetic testing of this patient were consistent with AS. In general, in approximately 75% of individuals with AS, the AS results from maternal 15q11-q13 deletion, which results in a more severe clinical phenotype than other genetic types of AS. AS is also caused by the *UBE3A* mutations (5–10%), paternal UPD of chromosome 15 (3–7%), or an imprinting center defect (2–3%). The remaining approximately 10% of patients with AS have no detectable genetic abnormality (Buiting et al., 2016; Beygo et al., 2019). Different genetic types in AS patients may show different phenotypes in performance (Yang et al., 2021).

Many aspects of the case presented here are consistent with previous reports of AS patients with the underlying cause of paternal UPD (Lossie et al., 2001; Poyatos et al., 2002; Thompson and Bolton, 2003; Tsai et al., 2004; Varela et al., 2004; Saitoh et al., 2005; Bonati et al., 2007; Depienne et al., 2009; Horváth et al., 2013; Luk and Lo, 2016), who generally had a lower prevalence of seizure, normal head circumference and absence of hypopigmentation (Lossie et al., 2001; Depienne et al., 2009; Horváth et al., 2013; Samanta, 2021). AS patients with UPD also have a higher risk of obesity than deletion type (Lossie et al., 2001; Poyatos et al., 2002; Varela et al., 2004; Saitoh et al., 2005; Brennan et al., 2015; Luk and Lo, 2016). A previous study found that children with UPD showed hyperphagic behavior and their weight increased significantly after the age of 2 years compared with children in the other genetic groups. These UPD children also had significantly higher birth weight than children with deletions or *UBE3A* mutations (Mertz et al., 2014). AS patients caused by UPD with no ataxia (Poyatos et al., 2002; Saitoh et al., 2005; Horváth et al., 2013; Luk and Lo, 2016) or no happy demeanor (Fridman et al., 2002; Varela et al., 2004; Bonati et al., 2007; Depienne et al., 2009; Luk and Lo, 2016) have also been reported. Moreover, it is notable that, despite one earlier study suggesting a decreased risk of ASD in AS in the absence of seizure (Thompson and Bolton, 2003), our case presented with autistic features without any history of seizure. This case adds to the pool of milder phenotypes in AS patients with UPD compared to a typical AS patients with deletions. A summary of the clinical features of our case and other AS cases with UPD is shown in Table 1. Of note, ataxic gait and frequent laughter/happy demeanor as consistent (100%) clinical features of patients with AS as claimed in a previous report (Williams et al., 2006) were not present in all AS patients with UPD in our literature review (Fridman et al., 2002; Poyatos et al., 2002; Thompson and Bolton, 2003; Varela et al., 2004; Saitoh et al., 2005; Bonati et al., 2007; Depienne et al., 2009; Horváth et al., 2013; Luk and Lo, 2016), supporting our hypothesis that AS resulting from UPD can manifest as a milder AS phenotype.

**TABLE 1:** Clinical features of Angelman syndrome patients with UPD of the patient in this study and patients from previous reports.

Clinical feature (s)	Lossie et al. (2001)	Poyatos et al. (2002) ^a^	Thompson and Bolton, (2003)	Varela et al. (2004) ^b^	Tsai et al. (2004)	Saitoh et al. (2005)	Bonati et al. (2007)	Depienne et al. (2009)	Horváth et al. (2013)	Luk and Lo, (2016)	Total number of literature patients	Patient in this study
Developmental delay/Intellectual disability	100%	4/4 (100%)	1/1 (100%)	5/5 (100%)	1/1 (100%)	3/3 (100%)	2/2 (100%)	1/1 (100%)	1/1 (100%)	6/6 (100%)	100%	**+**
Speech impairment	100%	4/4 (100%)	1/1 (100%)	5/5 (100%)	1/1 (100%)	3/3 (100%)	2/2 (100%)	1/1 (100%)	1/1 (100%)	6/6 (100%)	100%	**+**
Hand flapping	ND	3/3 (100%)	ND	ND	1/1 (100%)	ND	ND	ND	1/1 (100%)	ND	5/5 (100%)	**+**
Hyperactivity	ND	4/4 (100%)	1/1 (100%)	2/2 (100%)	ND	ND	ND	1/1 (100%)	ND	ND	8/8 (100%)	**-**
Abnormal EEG	ND	3/3 (100%)	ND	ND	ND	2/3 (66.7%)	2/2 (100%)	ND	ND	ND	7/8 (87.5%)	**-**
Frequent drooling	ND	3/3 (100%)	ND	3/4 (75%)	ND	ND	ND	ND	1/1 (100%)	ND	7/8 (87.5%)	-
Frequent laughter/Happy demeanor	ND	3/3 (100%)	1/1 (100%)	4/5 (80%)	1/1 (100%)	ND	1/2 (50%)	0/1 (0%)	1/1 (100%)	5/6 (83.3%)	16/20 (80%)	-
Abnormal/Wide based/Ataxia gait	ND	3/4 (75%)	1/1 (100%)	5/5 (100%)	1/1 (100%)	1/3 (33.3%)	2/2 (100%)	1/1(100%)	0/1 (0%)	3/6 (50%)	17/24 (70.8%)	**-**
Wide-spaced teeth	ND	ND	ND	2/4 (50%)	ND	ND	ND	1/1 (100%)	1/1 (100%)	ND	4/6 (66.7%)	**-**
Seizures	14/31 (45.2%)	4/4 (100%)	1/1 (100%)	3/5 (60%)	0/1 (0%)	1/3 (33.3%)	1/2 (50%)	0/1 (0%)	0/1 (0%)	4/6 (66.7%)	28/55 (50.9%)	-
Skin picking	ND	ND	ND	2/4 (50%)	ND	ND	ND	ND	ND	ND	2/4 (50%)	**-**
Weight (>95th centile, obesity)	9/27 (33.3%)	1/3 (33.3%)	ND	1/5 (20%)	0/1 (0%)	1/3 (33.3%)	0/2 (0%)	ND	ND	2/6 (33.3%)	14/47 (29.8%)	**+**
Hyperphagia	ND	1/3 (33.3%)	ND	1/4 (25%)	ND	ND	ND	ND	ND	ND	2/7 (28.6%)	**-**
Hypopigmentation	4/20 (20%)	1/3 (33.3%)	0/1 (0%)	ND	1/1 (100%)	2/2 (100%)	ND	ND	0/1 (0%)	1/6 (16.7%)	9/34 (26.5%)	**-**
Microcephaly (<5th centile)	4/27 (14.8%)	3/3 (100%)	ND	1/5 (20%)	ND	0/2 (0%)	0/2 (0%)	0/1 (0%)	0/1 (0%)	1/6 (16.7%)	9/47 (19.1%)	**-**
Hypotonia	ND	ND	ND	1/5 (20%)	ND	ND	ND	ND	0/1 (0%)	ND	1/6 (16.7%)	**-**
Mean Age to sit (months)	11 months	ND	6 months	ND	ND	ND	6 months	ND	ND	ND	** **	7 months
Mean Age to walk (years)	2.6 years	5.125 years	2 years	ND	3 years	2.9 years	2.9 years	ND	ND	ND	** **	2.25 years

a Excluding the recorded 12 patients from Lossie et al. (2001).

b We excluded the records of 4 patients from Lossie et al. (2001) and four of the five UPD patients reported in Varela et al. (2004) that were previously reported by Fridman et al. (2002).

+, present; −, absent; EEG, electroencephalogram; ND, not determined; UPD, uniparental disomy.

Previous studies have reported a wide range (1%–100%) of prevalence of ASD in AS caused by deletion, imprinting center defect, UPD or *UBE3A* mutations. The wide variety of percentages of ASD incidence in AS patients may be due to the small cohorts in such studies, which ranged from 4 to 93 AS cases (Chan et al., 1993; Steffenburg et al., 1996; Peters et al., 2004; Trillingsgaard and ØStergaard, 2004; Sahoo et al., 2006; Bonati et al., 2007; Mertz et al., 2014; Wink et al., 2015). The findings of the studies of ASD in AS are summarized in Table 2. However, many of the AS cases in these studies did not meet the full diagnostic criteria of ASD and lacked genetic verification for the diagnoses of AS. These studies mostly used standardized observation schedules such as ADOS for ASD diagnosis, however the ADOS is not a valid measure for children with mental ages below 18–24 months. Thus social and communication deficits in individuals with AS may be qualitatively different from these phenotypes in individuals with ASD. Among the studies of ASD in AS, Peters et al. and Bonati et al. reported two cases of AS with UPD who met the autism criteria from a total of 4 cases of AS with UPD in their studies. Furthermore, there is little information about the prevalence of AS in patients with ASD. Schroer et al. reported one AS case (a maternal 15q11-q13 deletion) among 100 patients with ASD (1%) and Depienne et al. reported two AS cases in a study of 552 cases with ASD (0.36%), one with a deletion and the other with UPD. Another previous study reported a prevalence of ASD in PWS individuals of 26.7% (Bennett et al., 2015). A systematic review found that ASD occurred more frequently in PWS patients with maternal UPD (35.3%) than those with deletion (18.5%) (Bennett et al., 2015). Although we found no cases of PWS or AS in our 100 patients with ASD, there is sufficient evidence for the occurrence of ASD in AS (Table 2), indicating that MS-PCR screening should be considered for AS and PWS in ASD patients, especially in patients with severe speech impairment and obesity (Lossie et al., 2001; Poyatos et al., 2002; Mertz et al., 2014; Luk and Lo, 2016). MS-PCR analysis is an easy and rapid method to screen for these syndromes simultaneously. MS-PCR analysis can detect more than 99% of affected individuals with PWS and approximately 80% of affected individuals with AS, including those with deletion, imprinting center defect or UPD (Beygo et al., 2019). However, further investigations such as haplotype analysis are needed to elucidate the underlying genetic mechanisms of disease for genetic counseling. The recurrence risk of having AS to the siblings of an affected child depends on the pathogenic mechanism. The risk to siblings is less than 1% if the affected child has a deletion or UPD, but up to 50% if the affected child has an imprinting center defect or a mutation of the *UBE3A* gene (Beygo et al., 2019).

**TABLE 2:** Summary of the studies of ASD in Angelman syndrome patients.

Author	Angelman syndrome (AS) patients					ASD diagnostic criteria	ASD frequency in AS patients	
	Total	Deletion	UPD	*UBE3A* mutation	ICD		Total	UPD
Chan et al. (1993)	93	60	3	ND	ND	Unknown	1/93 (1.1%)	ND
Steffenburg et al. (1996)	4	2	ND	ND	ND	DSM-III-R, DSM-IV, CARS, ABC, ADI-R	4/4 (100%)	ND
Peters et al. (2004)	19	16	2	1	-	DSM-IV, ADOS, ADI-R	8/19 (42.1%)	1/2 (50%)
Trillingsgaard and ØStergaard, (2004)	16	16	—	—	—	ADOS	13/16 (81.25%)	0
Sahoo et al. (2006)	22	22	—	—	—	ADOS, ADI-R	11/22 (50%)	0
Bonati et al. (2007)	23	8	2	7	6	DSM-IV, ADOS, ADI-R	18/23 (78.3%)	1/2 (50%)
Mertz et al. (2014)	39^a^	30^b^	5	4	—	ADOS	31/39 (79.5%)	5/9 (55.5%) (UPD or *UBE3A* mutation)
Wink et al. (2015)	12	4	3	4	1	ADOS, ADI-R	10/12 (83.3%)	ND

Note: Studies with less than three patients not included.

a 7 (all with deletion) of 39 patients were previously reported in Trillingsgaard and ØStergaard (2004).

b Excluding 3 patients with atypically large deletions.

ABC, Autism Behavior Checklist; ADI-R, Autism Diagnostic Interview-Revised; ADOS, Autism Diagnostic Observation Schedule; AS, Angelman syndrome; ASD, autism spectrum disorder; CARS, Childhood Autism Rating Scale; DSM, Diagnostic and Statistical Manual of Mental Disorders; ICD, imprinting center defect; ND, not determined; UPD, uniparental disomy.

In conclusion, we report an atypical AS child with autistic features and obesity caused by UPD of chromosome 15. Microarray analysis first identified the absence of heterozygosity of the q arm of chromosome 15. Subsequently, MS-PCR confirmed AS in this patient and microsatellite haplotype analysis revealed that he had received both chromosomes 15 from his father, which confirmed paternal UPD, leading to a final diagnosis of AS with UPD, with clinical features milder than a typical AS patient. Our findings suggest that a PWS/AS investigation may be useful in ASD cases with unknown etiology, as finding the underlying cause of ASD can lead to better genetic counseling for the family of the patient.

## Data Availability

The original contributions presented in the study are included in the article/Supplementary Material, further inquiries can be directed directed to the first author or corresponding author.
