# Supplementary material for: Case Report: An Atypical Angelman Syndrome Case With Obesity and Fulfilled Autism Spectrum Disorder Identified by Microarray
Source: Front Genet. 2021 Sep 22;12:755605. doi: 10.3389/fgene.2021.755605 (PMC8494305; doi:10.3389/fgene.2021.755605)
Supplement: Supplementary file 1 [file DataSheet1.PDF]

Supplementary Material

Case report: An Atypical Angelman Syndrome Case with Obesity and Fulfilled Autism Spectrum Disorder Identified by Microarray

Case Presentation

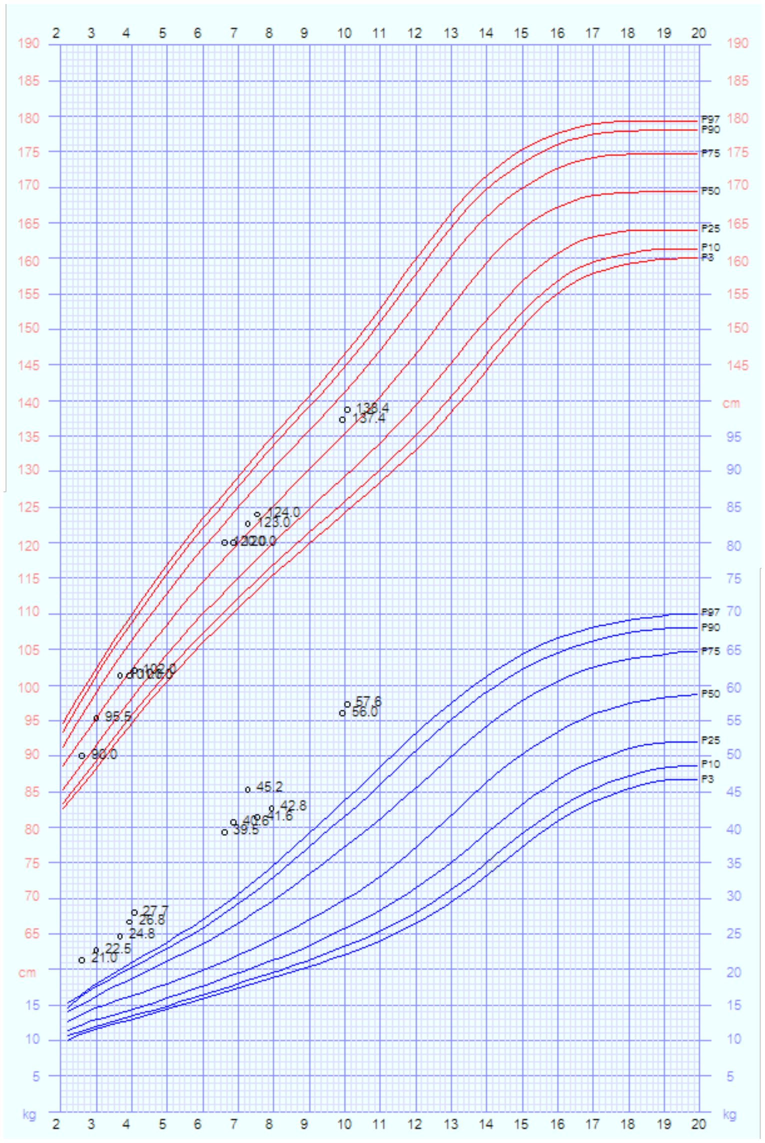

Supplementary Figure 1. Height and weight curves (~ 2-10 years of age) of the Angelman syndrome patient with ASD according to Thai children standard curves.

Supplementary Table 1. Autism Diagnosis Observation Schedule (ADOS)-Module 1 results of the patient at 5 years of age*.

Group	Score	Interpretation
**Communication**		
Frequency of Vocalization Directed to Others	1	
Stereotyped/Idiosyncratic Use of Words or Phrases	0	
Use of Other's Body to Communicate	1	
Pointing	2	
Gestures	0	
**Communication Total**	**4**	**Autism**
(Autism cut-off = 4; autism spectrum cut-off = 2)		
**Reciprocal Social Interaction**		
Unusual Eye Contact	2	
Facial Expressions Directed to Others	1	
Shared Enjoyment in Interaction	0	
Showing	2	
Spontaneous Initiation of Joint Attention	2	
Response to Joint Attention	0	
Quality of Social Overtures	1	
**Social Interaction Total**	**8**	**Autism**
(Autism cut-off = 7; autism spectrum cut-off = 4)		
**Communication Total + Social Interaction Total**	**12**	**Autism**
(Autism cut-off = 12; autism spectrum cut-off = 7)		
**Play**		
Functional Play With Objects	2	
Imagination/Creativity	2	
**Play Total**	**4**	
**Stereotyped Behaviors and Restricted Interests**		
Unusual Sensory Interest in Play Material/Person	0	
Hand and Figure and Other Complex Mannerisms	1	
Unusually Repetitive Interests or Stereotyped Behaviors	0	
**Stereotyped Behaviors and Restricted Interests Total**	**1**	
**ADOS Classification: Autism**		

* The patient was assessed one time using ADOS-Module 1 under a valid license of JW (co-author) for developing a Thai version of the ADOS.

Materials and Methods

Methylation-specific polymerase chain reaction (MS-PCR) analysis

The DNA samples were treated with bisulfate using a process described previously (Kubota et al., 1997) with modifications, briefly as follows: two micrograms of the genomic DNA in a volume of 50 µl of PCR reaction were denatured and modified by NaOH, hydroquinone and sodium bisulfate. The mixture was incubated at 55°C overnight and the modified DNA was then purified by an Illustra Blood GenomicPrep Mini Spin Kit (GE Healthcare). The DNA was desulphonated by adding NaOH to a final concentration of 0.3 M and the mixture incubated at room temperature for 10 minutes. The modified DNA was precipitated with absolute ethanol and resuspended in sterile water. The modified DNA sample was used as a template in the following MS-PCR. In addition, untreated DNA was used as an additional control to confirm complete sodium bisulfate conversion.

Supplementary Table 2. Primers used for methylation-specific PCR.

Primer set	Primer sequence (5'-> 3')	Product size (bp)	References
SNRPN-MF (Met)	TAAATAAGTACGTTTGCGCGGTC	174 bp	Kubota et al., 1997
SNRPN-MR (Met)	AACCTTACCCGCTCCATCGCG		
SNRPN-PF (Unmet)	GTAGGTTGGTGTGTATGTTTAGGT	100 bp	
SNRPN-PR (Unmet)	ACATCAAACATCTCCAACAACCA		
Alternative-MF (Met)	TCGATGGTATTTTGTTCGTTCGTATTGGGGCGC	152 bp	Hussain Askree et al., 2011
Alternative -MR (Met)	CCATATCCCTTACCCACTACGATTACCCCG		
Alternative -PF (Unmet)	TTGATGGTATTTTGTTTGTTTGTATTGGGGTGT	92 bp	
Alternative -PR (Unmet)	ACCACAGACACCCACAATAAAACCTATCACA		
SNRPNF*	GGAGGGAGCTGGGACCCC	220 bp	Hussain Askree et al., 2011
SNRPNR*	GAAGCCACCGGCACAGCT		

* Primers for untreated DNA to confirm complete sodium bisulfate conversion. Met, methylated allele; Unmet, unmethylated allele; P, paternal; M, maternal; F, forward primer; R reverse primer

Haplotype analysis of paternal UPD

To investigate either paternal or maternal UPD in the proband with autism, six microsatellite markers (D15S1012, D15S643, D15S983, D15S979, D15S657 and D15S966) located along the q arm of chromosome 15 were selected from the GenBank database. The details of the microsatellite markers and primers used for haplotype analysis are shown in Supplementary Table 2.

For haplotype analysis using capillary electrophoresis, all PCR reactions were carried out in a reaction volume of 10 µl containing 50 ng genomic DNA, 1.25 mM $MgCl_2$, 0.2 mM dNTP, 0.1 µM each of Carboxyfluorescein (FAM)-tagged forward primer and reverse primer, 1X PCR buffer and 0.5 unit of Taq DNA polymerase (Invitrogen). The PCR cycling process consisted of an initial denaturation at 95°C for 5 min, followed by 25 cycles at 95°C for 1 min, annealing at 65°C for 1 min, 72°C for 1 min, and final extension at 72 °C for 10 min. The samples were run on an ABI 3500 Genetic Analyzer and the data were analyzed using GeneMapper 3.7 software (Applied Biosystems).

Supplementary Table 3. Primers used for haplotype analysis.

**Microsatellite marker**	**Location**		**Repeat**	**Size range (bp)**	**Direction**	**Primer sequence (5'-> 3')**
	**Marshfield map (cM)**	**Chromosome location**				
D15S1012	35.95	15q14	CA	160-174	Forward	CAACAAGAACGAAACTGTCA
					Reverse	AGCCTTAAGTTCCTGGACTC
D15S643	52.33	15q22.2	TCTA	195-223	Forward	ATACCTGGAGTCCTTGGTCC
					Reverse	AACAGCTTTAAAACCTCAATGC
D15S983	66.90	15q23	CA	111-137	Forward	TCTGAAACGATGGGCTG
					Reverse	AAGGTGATTCCGTCCCTG
D15S979	83.40	15q25.3	CA	135-167	Forward	TGCTGCCCAACATCCT
					Reverse	CAGTGCTACATCCACGGAA
D15S657	104.86	15q26.2	TAGA	330-360	Forward	TCTACATTGGACAGAAATGGG
					Reverse	GATACACATTCTGATTCATGCG
D15S966	112.58	15q26.3	CA	210-252	Forward	TGCTGCTCACGAACTTTT
					Reverse	CCTCTTGGGAACTGTGTAGTATT

Results

(A)

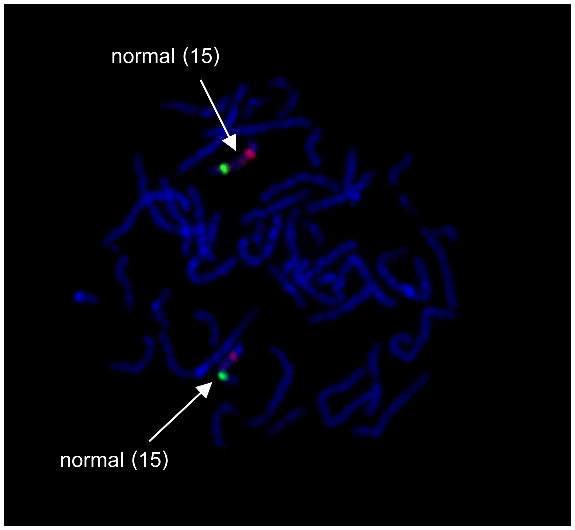

(B)

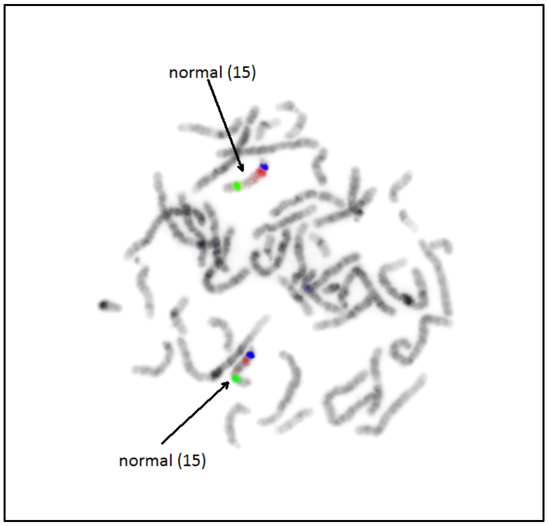

Supplementary Figure 2. FISH analyses of a proband with autism showing normal pattern in both chromosomes 15. (A) Metaphase FISH using a triple probe mix of Vysis Prader-Willi/Angelman Region Probe - LSI D15S10 SpectrumOrange at 15q11.2, CEP 15 (D15Z1) SpectrumAqua at 15p11.2, and PML SpectrumGreen Probe at 15q22. (B) The same metaphase FISH with phase-contrast.

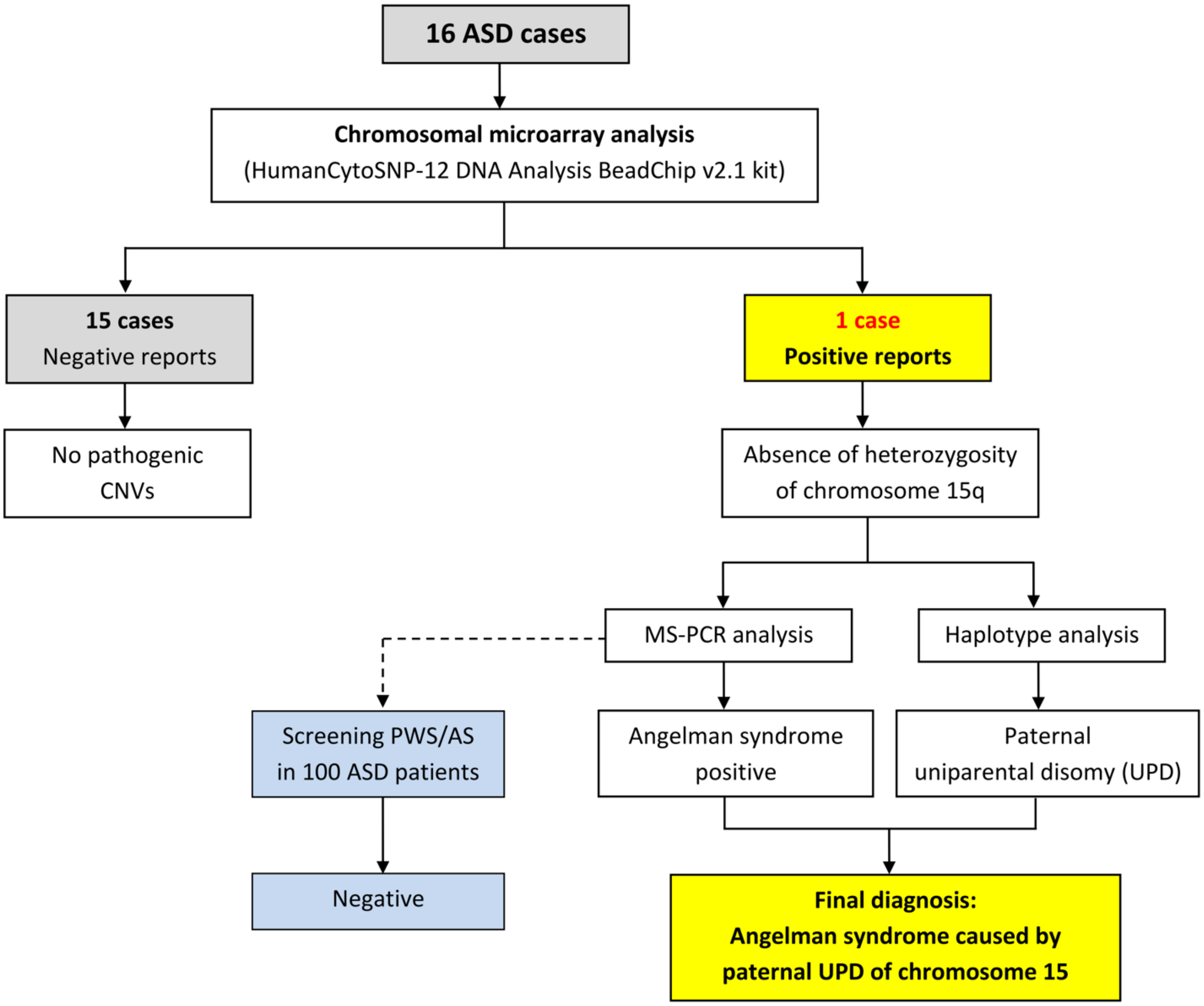

Supplementary Figure 3. Workflow summary of overall study results. ASD, autism spectrum disorder; CNVs, copy number variations; AS, Angelman syndrome; PWS, Prader-Willi syndrome; MS-PCR analysis, methylation-specific polymerase chain reaction analysis.

References

Hussain Askree, S., Hjelm, L. N., Ali Pervaiz, M., Adam, M., Bean, L. J. H., Hedge, M., et al. (2011). Allelic dropout can cause false-positive results for Prader-Willi and Angelman syndrome testing. *J. Mol. Diagn.* 13, 108–112. doi:10.1016/j.jmoldx.2010.11.006.

Kubota, T., Das, S., Christian, S. L., Baylin, S. B., Herman, J. G., and Ledbetter, D. H. (1997). Methylation-specific PCR simplifies imprinting analysis. *Nat. Genet.* 16, 16–17. doi:10.1038/ng0597-15.
